# Description and validation of a spectrum score method to measure antimicrobial de-escalation in healthcare associated pneumonia from electronic medical records data

**DOI:** 10.1186/s12879-015-0933-9

**Published:** 2015-04-25

**Authors:** Karl Madaras-Kelly, Makoto Jones, Richard Remington, Christina Caplinger, Benedikt Huttner, Matthew Samore

**Affiliations:** Pharmacy Service, Boise Veterans Affairs Medical Center and College of Pharmacy, Idaho State University, T111, 500 W Fort Street, Boise, 83702 USA; IDEAS Center, VA Salt Lake City Health Care System and Division of Epidemiology Health Care System and Division of Epidemiology, University of Utah, 500 Foothill Drive, Salt Lake City, 84148 UT USA; Research Service, Boise Veterans Affairs Medical Center and Quantified Inc, T111, 500 W Fort Street, Boise, 83702 USA; Research Fellow, Pharmacy Service, Boise Veterans Affairs Medical Center, T111, 500 W Fort Street, Boise, 83702 USA; Infection Control Programme & Division of Infectious Diseases, Faculty of Medicine, Geneva University Hospitals, Rue Gabrielle Perret- Gentil 4, Geneva, 1211 Switzerland; IDEAS Center, VA Salt Lake City Health Care System and Division of Epidemiology, University of Utah, 500 Foothill Drive, Salt Lake City, UT 84148 USA

**Keywords:** Antimicrobial de-escalation, Antimicrobial spectrum, Healthcare-associated pneumonia, Spectrum score, Multi-drug resistance, Broad-spectrum antimicrobials, Humans

## Abstract

**Background:**

Comparison of antimicrobial de-escalation rates between healthcare settings is problematic. To objectively and electronically measure de-escalation a method based upon the spectrum of antimicrobial regimens administered (i.e., spectrum score) was developed.

**Methods:**

A Delphi process was used to develop applicable concepts. Spectrum scores were created for 27 antimicrobials based upon susceptibility for 19 organisms. National VA susceptibility data was used to estimate microbial spectrum. Susceptibility estimates were converted to ordinal scores, and values for organisms with multi-drug resistance potential were weighted more heavily. Organism scores were summed to create antibiotic-specific spectrum scores and extended mathematically to score multi-antimicrobial regimens. Vignettes were created from antimicrobial regimens administered to 300 patients hospitalized with pneumonia. Daily spectrum scores were calculated for each case. Hospitalization day 4 scores were subtracted from day 2 scores (i.e., spectrum score ∆). A positive spectrum score ∆ defined de-escalation. Experts ranked each pneumonia case on a 7-point Likert scale (Likert >4 indicated de-escalation). Spectrum score ∆s were compared to expert review. Findings were used to identify score deficiencies. Next, 40 pairs of cases were modified to include antimicrobial administration routes. Each pair contained almost similar regimens; however, one contained oral (PO) the other only intravenous (IV) antimicrobials on day 4 of therapy. Experts reviewed cases as described. Spectrum score ∆ credits to account for PO conversion were derived from the mean paired differences in Likert Score. De-escalation status was evaluated in 100 vignettes containing antimicrobial route by different experts and compared to the modified method.

**Results:**

Initial sensitivity and specificity of the spectrum score ∆ to detect expert classified de-escalation events was 86.3 and 96.0%, respectively. In paired cases, the mean (± SD) Likert score was 5.0 (1.5) and 4.6 (1.5) for PO and IV (P = 0.002), respectively. To improve de-escalation event detection, two credits were added to spectrum score ∆s based upon the percentage of antimicrobials administered PO on day 4. The final method, exhibited sensitivity and specificity to detect expert classified de-escalation events of 96.2 and 93.6%, respectively.

**Conclusions:**

The final spectrum score method exhibited excellent agreement with expert judgments of de-escalation events in pneumonia.

**Electronic supplementary material:**

The online version of this article (doi:10.1186/s12879-015-0933-9) contains supplementary material, which is available to authorized users.

## Background

In response to increasing antimicrobial resistance, professional and governmental organizations recommend that hospital-based programs practice antimicrobial stewardship [1-3]. Antimicrobial stewardship refers to coordinated interventions to optimize the selection, dose, and duration of antimicrobial therapy, while limiting adverse events, cost, and antimicrobial resistance. Antimicrobial de-escalation is a fundamental operation of antimicrobial stewardship [1-5].

Antimicrobial de-escalation refers to a reduction in the antimicrobial “spectrum” of therapy, through discontinuation of antimicrobials providing activity against non-pathogenic flora or changing antimicrobials to limit coverage to targeted pathogens once a patient is clinically stable [1-5]. Stopping antimicrobials with overlapping spectrum or, discontinuing antimicrobials if an infection is unlikely may also be considered antimicrobial de-escalation [5,6]. In studies, de-escalation has been defined and measured subjectively based upon variable qualitative assessments [5]. Further, manual chart review is required to determine if antimicrobial de-escalation has occurred. Comparison of antimicrobial de-escalation practice is limited due to a lack of objective measurement criteria and automated measurement methods.

We developed a method to measure antimicrobial de-escalation, which was based upon the spectrum of antimicrobial regimens administered that can be applied to electronic medical records data (i.e., spectrum score method). The methodological framework was based upon the opinions of antimicrobial stewards for concepts relevant to antimicrobial de-escalation, which were obtained through a Delphi process. The method was developed to estimate facility-level de-escalation rates with data from the U.S. Veterans Healthcare Administration (VA) Computerized Patient Record System. A description of the Delphi panel findings and a preliminary overview of the spectrum score method have been reported [7].

The purpose of this manuscript is to provide a detailed description of the spectrum score method, including: the approach to assignment of spectrum scores to antimicrobials, application of the scores to electronic medical records data to generate spectrum scores for antimicrobial regimens; refinement and validation of the method to measure antimicrobial de-escalation in patients with Healthcare Associated Pneumonia (HCAP). HCAP was selected for evaluation because broad-spectrum empirical antimicrobial therapy and de-escalation are indicated [8].

## Methods

The spectrum score method involves assignment of a numerical score to each calendar day of antimicrobial therapy administered during hospitalization based on the microbial spectrum of the regimen. Antimicrobial de-escalation is quantified by subtracting the regimen’s spectrum score for day 4 of hospitalization from a baseline score obtained for antimicrobials administered on day 2 of hospitalization (i.e., spectrum score ∆). A positive spectrum score ∆ suggests that microbial spectrum coverage has been narrowed and that de-escalation has occurred.

### Assignment of spectrum score to antimicrobials

The approach to assigning a numerical spectrum score to antimicrobial regimens has recently been summarized [7]. First, national VA Corporate Data Warehouse (CDW) susceptibility data for organisms and antimicrobials tested during years 2008–2012 were used to estimate antimicrobial susceptibility if available. The VA CDW includes culture and susceptibility results for 7 million veterans who receive care in 152 medical centers. Percent susceptibility was calculated for individual antimicrobial-organism pairs utilizing one isolate per patient per year. Next, remaining organism-antimicrobial pairs were categorized as possessing no intrinsic activity (NA), no VA susceptibility data available (ND), or further confirmation of susceptibility estimates required. NA classifications were based upon current FDA approved prescribing information, Clinical Laboratory Standards Institute (CLSI) standards for susceptibility testing, and tertiary references [9-42]. Current CLSI reporting recommendations were applied preferentially. For example, the CLSI-based susceptibility of *Staphylococcus aureus* to oxacillin was used to populate the susceptibility values for other β-lactams (except ceftaroline) with activity against *Staphylococcus aureus*. Organism-antimicrobial pairs without approved susceptibility methods, but suppressed results, were handled individually; where possible findings were cross-referenced with susceptibility results identified through literature sources.

Assignment of scores to organism-antimicrobial pairs without susceptibility data was directed by several data sources, and in some cases more than one data source was used. Primary literature was evaluated for susceptibility studies. Preference was given to results from studies which included U.S. based isolates, referenced CLSI methods, and recent studies. In some cases, CLSI documents provided estimates of susceptibility, in other cases, if recent product labeling indicated that the antimicrobial possessed *in vitro* activity against > 90% of the isolates tested in clinical trials, a value of 90% was assigned. Infrequently, suitable references could not be identified, and investigator opinion was utilized to assign susceptibility. To enhance generalizability and apply susceptibility data to be more representative of different practice environments, percentage susceptibilities were converted into quintiles ranging from 0 points for susceptibilities of < 20% to 4 points for susceptibility of 80-100%. Assignment of ordinal scores to organism-antimicrobial pairs without VA susceptibility data was performed independently by two investigators (KM, BH) with adjudication of discrepancies by a 3rd investigator (MJ).

### Spectrum score adjustment for intrinsically resistant organisms

Delphi panelists indicated that antimicrobials covering organisms with high potential for developing resistance should receive extra weight in the spectrum score [7]. Consistent with panelist preferences, ordinal susceptibility values for organism-antimicrobial pairs involving *Staphylococcus aureus*, *Enterococcus faecium, Escherichia coli*, *Klebsiella spp.,* and *Acinetobacter spp.* were multiplied by a factor of 1.25, and *Pseudomonas aeruginosa* was multiplied by a factor of 1.75. Domains for each organism’s weighted or un-weighted scores were added to create a composite spectrum score for each antimicrobial on a 0–60 spectrum of activity scale.

### Calculation of spectrum scores for combination antimicrobial therapy

To account for overlapping coverage in combination regimens, the following approach was taken. First, if none of the regimen antimicrobials possessed activity against a species, a zero was assigned to the organism-combination regimen pair. If one of the regimen antimicrobials possessed activity against a species, the active antimicrobial susceptibility was used to populate the organism combination regimen pair. For other regimens where a combination of antimicrobials possessed activity against a species, the proportion of organisms susceptible to a multi-antimicrobial regimen was estimated as one minus the joint probability of resistance to all regimen antimicrobials, assuming that antibiotic susceptibilities were independent [7]. Spectrum scores were then computed identically to individual antimicrobial regimens. Table 1 illustrates the steps involved in generating spectrum score values for an individual and combination antimicrobial regimens.

**Table 1:** **Conceptual illustration of spectrum score calculation for an antimicrobial regimen**

**Step 1: Populate susceptibility percentage for organism domains, convert values to ordinal scale (ordinal values shown in parentheses)** ^**A,B,C,D**^						
**Organism domain**	**Vancomycin susceptibility (%)**	**Vancomycin ordinal score**	**Cefepime susceptibility (%)**	**Cefepime ordinal score**	**Vancomycin + cefepime joint susceptibility** ^**B**^	**Vancomycin + cefepime joint ordinal score**
*Staphylococcus aureus*	99.3	4	51.2	2	99.6	4
*Streptococcus pneumoniae*	98.2	4	92.5	4	99.9	4
*Enterococcus faecium*	18.4	0	NA	0	18.4	0
*Enterococcus faecalis*	94.7	4	NA	0	94.7	4
*Escherichia coli*	NA	0	92.4	4	92.4	4
*Klebsiella spp.*	NA	0	84.5	4	84.5	4
Other enterobacteriaceae^C^	NA	0	91.3	4	91.3	4
*Pseudomonas aeruginosa*	NA	0	78.0	3	78.0	3
*Acinetobacter spp.*	NA	0	42.6	2	42.6	2
*Stenotrophomonas spp.*	NA	0	36.6	1	36.6	1
*Haemophilus influenzae*	NA	0	95.4	4	95.4	4
*Bacteroides spp.*	NA	0	NA	0	NA	0
*Legionella spp.*	NA	0	NA	0	NA	0
*Mycoplasma spp.*	NA	0	NA	0	NA	0
**Step 2: Weight susceptibilities for coverage against intrinsically resistant organisms, sum organism domain scores to create antibiotic regimen spectrum score** ^**E**^						
**Organism domain**	**Vancomycin weighted ordinal score**		**Cefepime weighted ordinal score**		**Vancomycin + cefepime weighted ordinal score**	
*Staphylococcus aureus*	5		2.5		5	
*Streptococcus pneumoniae*	4		4		4	
*Enterococcus faecium*	0		0		0	
*Enterococcus faecalis*	4		0		4	
*Escherichia coli*	0		5		5	
*Klebsiella spp.*	0		5		5	
Other enterobacteriaceae^C^	0		4		4	
*Pseudomonas aeruginosa*	0		5.25		5.25	
*Acinetobacter spp.*	0		2.5		2.5	
*Stenotrophomonas spp.*	0		1		1	
*Haemophilus influenzae*	0		4		4	
*Bacteroides spp.*	0		0		0	
*Legionella spp.*	0		0		0	
*Mycoplasma spp.*	0		0		0	
**Spectrum Score**	**13.0**		**33.25**		**39.75**	

Spectrum Score Calculations for Individual and Combination Antibiotic Regimens. ^A^Values populated with susceptibility data. Susceptibility estimates for combinations where all antimicrobials possessed activity against the species obtained by calculating one minus the joint probability of resistance to all antibiotics in the regimen, assuming that susceptibility was independent for each antibiotic. ^B^Other enterobacteriaceae included: *Citrobacter spp., Enterobacter spp., Morganella spp., Proteus spp., Providencia spp., Serratia spp*. ^C^NA = No intrinsic activity. ^D^Ordinal values were 0 for no intrinsic bacterial activity or susceptibility < 20%, 1 for > 20 but < 40%, 2 for >40 but < 60%, 3 for >60 but < 80%, 4 > 80 %. ^E^A weight of 1.25 was applied to ordinal domain values for *Staphylococcus aureus, Escherichia coli, Klebsiella spp., Acinetobacter spp., Enterococcus faecium,* and a weight of 1.75 was applied to spectrum score values for *Pseudomonas aeruginosa.*

### Application of the spectrum score method to measure de-escalation

Inpatient antimicrobial administration within the VA is documented utilizing Bar Code Medication Administration (BCMA) technology. For each administered dose of antimicrobial, data regarding the dose and route of administration are recorded electronically with a time stamp [43]. Antimicrobial use data was obtained for a VA-wide cohort (years 2008–2012) of inpatient admissions with HCAP [8,44]. Each systemically administered antimicrobial the patient received during each calendar day of admission was extracted from the CDW [45]. As combination antimicrobial therapy was commonly administered, data were “smoothed” to prevent over and under-calculation of spectrum scores on days when different antimicrobials were simultaneously being initiated or discontinued. Smoothing was accomplished by inferring the intent to treat from antimicrobial administration data. Skip days, where antibiotics that were administered less frequently than once daily, were filled when between adjacent calendar days where the same antibiotic was administered (e.g., vancomycin administered every 48 hours). Skip days were assigned the same antibiotic regimen as the adjacent calendar days. To avoid double counting antibiotics when an antibiotic regimen was switched, an antibiotic day was not counted when there was a change in a multidrug regimen from the current, previous, and next days, the antibiotic was given in the past two days but not in the next three, and the day did not fall on the beginning or end of an antibiotic treatment course. For example, switching from cefepime and vancomycin to levofloxacin and clindamycin on day 4 would appear as a four-drug regimen unless these rules are invoked, in which case only the latter regimen would be counted (Table 2).

**Table 2:** **Example of a prediction of antimicrobial de-escalation status by the spectrum score method**

**A. Daily antimicrobial (Route of administration) use data**					
**Antimicrobial/Day**	**Hospital day 1**	**Hospital day 2**	**Hospital day 3**	**Hospital day 4**	**Hospital day 5**
Clindamycin (Clm)				Clm (PO)	Clm (PO)
Levofloxacin (Lev)				Lev (PO)	Lev (PO)
Cefepime (Cpm)	Cpm(IV)	Cpm(IV)	Cpm (IV)	Cpm (IV)	
Vancomycin (Vm)	Vm (IV)	Vm (IV)	Vm (IV)	Vm (IV)	
Daily Regimen Spectrum Score	39.75	39.75	39.75	55.00	44.25
**B. Application of “smoothing” rules to daily antimicrobial use data**					
**Antimicrobial/Day**	**Hospital day 1**	**Hospital day 2**	**Hospital day 3**	**Hospital day 4**	**Hospital day 5**
Clindamycin (Clm)				Clm (PO)	Clm (PO)
Levofloxacin (Lev)				Lev (PO)	Lev (PO)
Cefepime (Cpm)	Cpm(IV)	Cpm (IV)	Cpm (IV)		
Vancomycin (Vm)	Vm (IV)	Vm (IV)	Vm (IV)		
Daily Regimen Spectrum Score	39.75	39.75	39.75	44.25	44.25
**C. Calculation of spectrum score ∆ (Day 2 spectrum score – Day 4 spectrum score)**					
**[Cefepime + vancomycin score]**	**–**	**[Clindamycin + levofloxacin score]**	**=**	**Spectrum score ∆**	
39.75	–	44.25	=	-4.5	
**D. Adjustment of spectrum score ∆ for PO antimicrobials administered on day 4**					
**Spectrum score ∆**	**+**	**Administration credit**	**Final spectrum score ∆ after PO adjustment**		
-4.5	+	6	1.5		

A. Example of antimicrobials administered on a daily basis during a 5 day hospitalization. Days 2 and 4 indicate baseline and de-escalation determination endpoints. Note that the daily antimicrobial regimen spectrum score increases on day 4 due to the addition of oral antimicrobials; however, IV antimicrobials are discontinued. B. Smoothing rules were applied to the daily antimicrobial administration data which results in a lower day 4 spectrum score. C. The spectrum score ∆ is negative suggesting that the spectrum of activity of is greater for the clindamycin + levofloxacin regimen than for the cefepime + vancomycin combination. D. The PO credit is applied to the case because 100% of the antimicrobials on day 4 were administered PO (6 points for regimens with ≥ 50 % of antimicrobials administered PO by day 4). The final spectrum score ∆ with PO credit is positive prediciting that a de-escalation event occurred.

### Spectrum score method refinement and validation

Refinement and validation of the spectrum score method was performed in three stages. First, 300 vignettes were created based on daily antimicrobial regimens obtained from a random sample of patients who met HCAP criteria [8,44]. Vignettes included antimicrobials administered on each calendar day of hospitalization and microbiology findings obtained in the first two days. Three antimicrobial stewards [mean (SD) 17.3 (0.7) years of experience] who were unfamiliar with the spectrum score method reviewed the vignettes. The stewards ranked each case on a 7-point Likert scale: de-escalation (score >4), no meaningful change in therapy (score =4), or escalation (score of < 4). Spectrum scores were determined for regimens administered on calendar days 2 and 4 of hospitalization; day 4 scores were subtracted from day 2 scores resulting in a spectrum score ∆ for each vignette. A positive spectrum score ∆ indicated de-escalation. Sensitivity and specificity of the sign of spectrum score ∆ to predict antimicrobial steward de-escalation opinion (i.e., reference standard) were calculated. To determine if varying the weight for coverage of resistant organisms impacted test characteristics of the method, spectrum score variants with differing weights (no weight, 1.25, 1.5, 1.75, 2.0, 3.0) for coverage of these organisms were calculated for the cases, and then compared to expert opinion in a similar manner.

**Figure 1:**
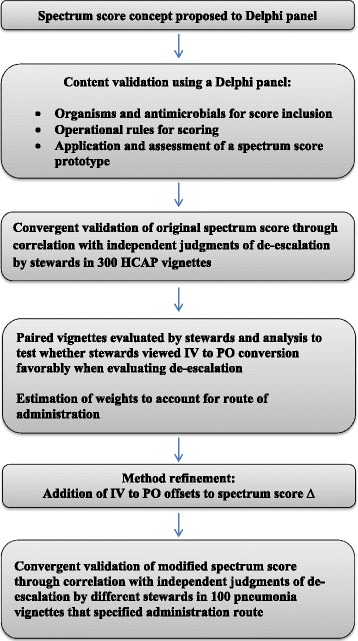
Overview of the process for development, refinement, and validation of the spectrum score method.

Based upon observations in the initial exercise, 40 pairs of vignettes were modified to include the administration route for each antimicrobial. Each pair of vignettes contained identical or almost identical antimicrobial regimens with the same spectrum; however, in one case the regimen on day 4 contained one or more antimicrobials administered orally (PO) whereas in the other case all antimicrobials were administered intravenously (IV) (i.e. amoxicillin versus ampicillin). These cases were evaluated by the same experts and compared to the spectrum score ∆ as described. Mean paired difference in Likert scores between the IV and PO cases was calculated for pairs in which the PO case had 50-100% PO therapy and for pairs with >0% but less than 50% PO therapy on day 4. A linear regression model relating mean Likert and spectrum score Δ for vignettes with 100% IV therapy on day 2 and day 4 was used to express the means of paired difference in Likert in spectrum score scale (paired Likert means divided by the slope). The mean values converted to the spectrum score scale provided additional credits to the spectrum score Δ to account for vignettes with minimal conversion to PO therapy (>0% but <50%) and greater or full conversion to PO therapy (50%-100%) on day 4. Finally, 100 vignettes were selected from the original 300 HCAP cases and the route of antimicrobial administration data was added. These vignettes were assessed for de-escalation status by three new antimicrobial stewards unfamiliar with the spectrum score method [mean (SD) 14.0 (5.2) years of experience] using the same 7-point Likert scale. Mean Likert scores were compared to the spectrum score ∆ credited for IV to PO conversion. Only vignettes with 100% IV therapy on day 2 were eligible for the credit. Test characteristics of the spectrum score method to predict antimicrobial de-escalation were performed as above. Table 2 illustrates application of the spectrum score method to measure de-escalation in a theoretical case, and Figure 1 summarizes the process for development, refinement, and validation of the spectrum score method. A procedure that can be used to apply the spectrum score method is available in Additional files 1 and 2.

This research complies with all Federal guidelines and VA policies relative to Human Subjects and research. The VA Puget Sound Healthcare System, University of Utah, and Idaho State University Human Subjects Committees approved the study and its conduct was in compliance with the Helsinki Declaration.

## Results

### Spectrum score calculations

Spectrum scores were determined for 14 organism domains which included 19 bacterial species and 27 categories of antimicrobials. Table 3 illustrates the ordinal susceptibility scores for organism-antimicrobial pairs. There were 513 possible organism-antimicrobial pairs for consideration of which 53% had sufficient VA susceptibility data, 6% were determined by applying CLSI-based rules, 28% were identified as possessing NA, and 15% required further confirmation or determination of susceptibility results with external sources. Suitable references could not be identified in a 3% of cases and domain scores were assigned by investigator opinion.

**Table 3:** **Ordinal susceptibility scores for antimicrobial-organism pairs included in the spectrum score**

	Anti-MRSA*					βL- Inhibitors**			Carbapenems & aztreonam***			Cephalosporins****			
	**Vanc**	**Lzld**	**Tig**	**Dapto**	**Ceftar**	**Pip/Taz**	**Tic/Clav**	**Amino-BLI**	**PSA Carb**	**Erta**	**Aztr**	**1st Gen Ceph**	**2nd Gen Ceph**	**3rd Gen Ceph**	**PSA Ceph**
**Gram positive organisms**															
*Staphylococcus aureus*	4Ϯ	4Ϯ	4Ϯ	4Ϯ	4κ	2¥	2¥	2¥	2¥	2¥	0I	2¥	2¥	2¥	2¥
*Streptococcus pneumoniae*	4Ϯ	4Ϯ	4κ	0Ψ	4κ	3¥	3¥	3¥	4Ϯ	4Ϯ	0I	3Ϯ	3Ϯ	4Ϯ	4Ϯ
*Enterococcus faecalis*	4Ϯ	4Ϯ	4Ϯ	4Ϯ	0¥	4Ϯ	4κ	4Ϯ	4Ϯ	0κ	0I	0¥	0¥	0¥	0¥
*Enterococcus faecium*	4Ϯ	4Ϯ	4Ϯ	4Ϯ	0¥	1Ϯ	1κ	1Ϯ	0Ϯ	0κ	0I	0¥	0¥	0¥	0¥
**Gram negative organisms**															
*Escherichia coli*	0I	0I	4Ϯ	0I	4κ	4Ϯ	4Ϯ	3Ϯ	4Ϯ	4Ϯ	4Ϯ	4Ϯ	4Ϯ	4Ϯ	4Ϯ
*Klebsiella spp.*	0I	0I	4Ϯ	0I	4κ	4Ϯ	4Ϯ	4Ϯ	4Ϯ	4Ϯ	4Ϯ	4Ϯ	4Ϯ	4Ϯ	4Ϯ
OtherEnterobacteriaceae	0I	0I	3Ϯ	0I	3κ	4Ϯ	4Ϯ	2Ϯ	4Ϯ	4Ϯ	4Ϯ	1Ϯ	2Ϯ	4Ϯ	4Ϯ
*Enterobacter spp.*	0I	0I	4Ϯ	0I	3κ	4Ϯ	3Ϯ	0Ϯ	4Ϯ	4Ϯ	4Ϯ	0Ϯ	1Ϯ	4Ϯ	4Ϯ
*Citrobacter spp.*	0I	0I	4Ϯ	0I	4κ	4Ϯ	4Ϯ	3Ϯ	4Ϯ	4Ϯ	4Ϯ	2Ϯ	3Ϯ	4Ϯ	4Ϯ
*Serratia spp.*	0I	0I	4Ϯ	0I	3κ	4Ϯ	4Ϯ	0Ϯ	4Ϯ	4Ϯ	4Ϯ	0Ϯ	0Ϯ	4Ϯ	4Ϯ
*Morganella spp.*	0I	0I	2Ϯ	0I	3κ	4Ϯ	3Ϯ	0Ϯ	4Ϯ	4Ϯ	4Ϯ	0Ϯ	0Ϯ	4Ϯ	4Ϯ
*Proteus spp.*	0I	0I	1Ϯ	0I	4κ	4Ϯ	4Ϯ	4Ϯ	4Ϯ	4Ϯ	4Ϯ	4Ϯ	4Ϯ	4Ϯ	4Ϯ
*Providencia spp.*	0I	0I	2Ϯ	0I	0Ψ	4Ϯ	4Ϯ	0	4Ϯ	4Ϯ	4Ϯ	0Ϯ	2Ϯ	4Ϯ	4Ϯ
*Pseudomonas aeruginosa*	0I	0I	0I	0I	0I	*4*Ϯ	4Ϯ	0I	4Ϯ	1Ϯ	4Ϯ	0I	0I	0Ϯ	4Ϯ
*Acinetobacter spp.*	0I	0I	4Ϯ	0I	0κ	3Ϯ	3Ϯ	3Ϯ	3Ϯ	0κ	0Ϯ	0Ψ	0Ψ	1Ϯ	3Ϯ
*Stenotrophomonas spp.*	0I	0I	3Ϯ	0I	0κ	3Ϯ	2Ϯ	0I	0Ϯ	0κ	0Ϯ	0Ψ	0Ψ	0Ϯ	1Ϯ
*Haemophilus influenzae*	0I	0κ	4κ	0I	4κ	*4¥*	4Ψ	4Ϯ	4Ϯ	4κ	4κ	3Ψ	4Ϯ	4Ϯ	4Ϯ
*Bacteroides spp.*	0I	0κ	4κ	0I	0I	4Ϯ	3Ω	4Ϯ	4Ϯ	4¥	0I	0I	0I	1Ϯ	0I
**Other organisms**															
Atypical organisms	0I	0κ	4κ	0I	0I	0I	0I	0I	0I	0I	0I	0I	0I	0I	0I
*Legionella spp.*	0I	0κ	4κ	0I	0I	0I	0I	0I	0I	0I	0I	0I	0I	0I	0I
*Mycoplasma spp.*	0I	0κ	4κ	0I	0I	0I	0I	0I	0I	0I	0I	0I	0I	0I	0I
	Penicillins*****		Miscellaneous******												
	**Amino PCN**	**Semi Synth PCN**	**PSAFQ**	**NON-PSAFQ**	**Amik**	**Gent or Tobra**	**Macro**	**Clinda**	**Tetra**	**TMP/SMX**	**Metro**	**Colistin**			
**Gram positive organisms**															
*Staphylococcus aureus*	0Ϯ	2Ϯ	2Ϯ	3Ϯ	4Ϯ	4Ϯ	1Ϯ	3Ϯ	4Ϯ	4Ϯ	0I	0κ			
*Streptococcus pneumoniae*	3Ϯ	3¥	4Ϯ	4Ϯ	0κ	0κ	3Ϯ	4Ϯ	3Ϯ	3Ϯ	0I	0κ			
*Enterococcus faecalis*	4Ϯ	0κ	3Ϯ	3Ϯ	0I	0I	0Ϯ	0¥	1Ϯ	0¥	0I	0κ			
*Enterococcus faecium*	0Ϯ	0κ	0Ϯ	0Ϯ	0I	0I	0Ϯ	0¥	1Ϯ	0¥	0I	0κ			
**Gram negative organisms**															
*Escherichia coli*	2Ϯ	0I	3Ϯ	3Ϯ	4Ϯ	4Ϯ	0I	0I	3Ϯ	3Ϯ	0I	4Ϯ			
*Klebsiella spp.*	0Ϯ	0I	4Ϯ	4Ϯ	4Ϯ	4Ϯ	0I	0I	4Ϯ	4Ϯ	0I	4Ϯ			
OtherEnterobacteriaceae	1Ϯ	0I	3Ϯ	3Ϯ	4Ϯ	4Ϯ	0I	0I	2Ϯ	4Ϯ	0I	2Ϯ			
*Enterobacter spp.*	0Ϯ	0I	4Ϯ	4Ϯ	4Ϯ	4Ϯ	0I	0I	4Ϯ	4Ϯ	0I	4Ϯ			
*Citrobacter spp.*	0Ϯ	0I	4Ϯ	4Ϯ	4Ϯ	4Ϯ	0I	0I	4Ϯ	4Ϯ	0I	4Ϯ			
*Serratia spp.*	0Ϯ	0I	4Ϯ	4Ϯ	4Ϯ	4Ϯ	0I	0I	0Ϯ	4Ϯ	0I	3Ϯ			
*Morganella spp.*	0Ϯ	0I	3Ϯ	2Ϯ	4Ϯ	4Ϯ	0I	0I	1Ϯ	3Ϯ	0I	1Ϯ			
*Proteus spp.*	3Ϯ	0I	3Ϯ	3Ϯ	4Ϯ	4Ϯ	0I	0I	0Ϯ	3Ϯ	0I	0κ			
*Providencia spp.*	0Ϯ	0I	2Ϯ	2Ϯ	4Ϯ	2Ϯ	0I	0I	0Ϯ	3Ϯ	0I	0Ψ			
*Pseudomonas aeruginosa*	0I	0I	3Ϯ	1Ϯ	4Ϯ	4Ϯ	0I	0I	0I	0I	0I	4Ϯ			
*Acinetobacter spp.*	0Ϯ	0I	3Ϯ	2Ϯ	4Ϯ	3Ϯ	0I	0I	2Ϯ	3Ϯ	0I	4Ϯ			
*Stenotrophomonas spp.*	0Ϯ	0I	4Ϯ	2Ϯ	2Ϯ	1Ϯ	0I	0I	3Ϯ	4Ϯ	0I	4Ϯ			
*Haemophilus influenzae*	3¥	0I	4Ϯ	4Ϯ	4κ	4κ	4Ϯ	0Ψ	4Ϯ	3Ϯ	0I	4κ			
*Bacteroides spp.*	0Ϯ	0I	2κ	2¥	0I	0I	0κ	3Ϯ	1κ	0κ	4Ϯ	0I			
**Other organisms**															
Atypical organisms	0I	0I	4κ	4κ	2κ	2κ	4κ	0κ	4κ	2κ	0I	0I			
*Legionella spp.*	0I	0I	4κ	4κ	4κ	0κ	4κ	0κ	4κ	4κ	0I	0I			
*Mycoplasma spp.*	0I	0I	4κ	4κ	0Ψ	0Ψ	4κ	0κ	4κ	0κ	0I	0I			

Special Notations:

Ϯ VA susceptibility data.

I No intrinsic activity.

Ψ Investigator opinion.

Ω Limited VA susceptibility data.

¥ Expert rule.

Κ Literature based.

Abbreviations:

*Anti-MRSA: Vanc = vancomycin; Lzld = linezolid; Tig = tigecycline; Dapto = daptomycin, Ceftar = ceftaroline.

**βL- Inhibitors: Pip/Taz = piperacillin/tazobactam; Tic/Clav = ticarcillin/clavulanate; AminoBLI = ampicillin/sulbactam, amoxicillin/clavulanate.

***Carbapenems and Aztreonam: PSACarba = imipenem, meropenem, Erta = ertapenem, Aztr = aztreonam.

****Cephalosporins: 1st Gen Ceph. = cefazolin, cephalexin; 2nd Gen Ceph = cefuroxime; 3rd Gen Ceph = ceftriaxone, cefotaxime, cefpodoxime; PSACeph = cefepime, ceftazidime.

*****Penicllins: AminoPCN = ampicillin, amoxicillin, penicillin; SemiSynthPCN = oxacillin, nafcillin.

******Miscellaneous: PSAFQ = ciprofloxacin or levofloxacin; NONPSAFQ = moxifloxacin, gemifloxacin; Amik = Amikacin; Gent or Tobra = gentamicin, tobramycin; Macro = erythromycin, azithromycin, clarithromycin; Clinda = clindamycin; Tetra = tetracycline, doxycycline; TMP/SMX = trimethoprim/sulfamethoxazole; Metro = metronidazole; Colistin = colistin.

### Spectrum score method refinement and validation

Findings for the initial validation exercise have recently been overviewed [7]. Briefly, there were 142 distinct antimicrobial regimens (79% combination regimens) administered in the 300 HCAP patient cases. Day 2 and 4 spectrum scores in the vignettes ranged from a minimum of 4.0 to a maximum of 60.0. The sensitivity and specificity of the spectrum score method to identify antimicrobial de-escalation events as determined by antimicrobial stewards was 86.3 and 96.0%, respectively. Adjustment of weights assigned for coverage of intrinsically resistant organisms did not improve the sensitivity and specificity of the spectrum score method beyond the weighting proposed by the Delphi panel, and spectrum score ∆ was more predictive of de-escalation events with application of these weights (P = 0.05, difference in area under the curve between weighted and un-weighted receiver operator curves). Upon inspection, it appeared that in select vignettes stewards inferred that a patient received a PO antimicrobial on day 4 even though the route of antimicrobial administration was not stated (e.g., ceftriaxone administered on day 2 followed by cefpodoxime administered on day 4). Despite many of these regimens possessing similar spectrum scores (i.e., spectrum score for both ceftriaxone or cefpodoxime was 25.25) , experts scored these vignettes higher if regimens contained antimicrobials with PO dosage forms available compared to regimens with IV antibiotics on day 4 (e.g., ceftriaxone on day 2 and day 4).

In the second vignette exercise, the mean (± SD) Likert score for paired vignettes containing identical or nearly identical antimicrobials with the same spectrum but differing routes of administration by day 4 was 5.0 (1.5) and 4.6 (1.5) for PO and IV cases (*P* = 0.002), respectively. The mean (± SD) paired difference in Likert scores was 0.44 (0.50) for pairs where the percentage of PO therapy in the PO case was 50-100% by day 4 (*P* < 0.001) and 0.24 (0.68) for pairs where the percentage of PO therapy was > 0% but < 50% in the PO case by day 4 (*P* = 0.12). Based on the linear relationship between mean Likert score and weighted spectrum score ∆ (slope = 0.072, *P* < 0.001) for vignettes with 100% IV therapy on day 2 and day 4, the paired mean difference in Likert score for vignettes with 50-100% PO therapy on day 4 was equivalent to approximately 6 spectrum score points, and was equivalent to approximately 3 spectrum score points for >0% but <50% PO therapy on day 4. Adding the two credits to spectrum score ∆s for applicable regimens improved detection of de-escalation events in the set of paired vignettes as classified by stewardship experts.

In the final exercise, the sensitivity and specificity of the spectrum score method, including the credit for PO antimicrobials to predict de-escalation events was 96.2 (95% CI >88.6%) and 93.6% (95% CI >84.3%), respectively. Antimicrobial stewards identified de-escalation, no meaningful change in therapy, or escalation in 53, 41, and 6% of vignettes reviewed whereas, the spectrum score method with credits identified de-escalation, no meaningful change in therapy, or escalation by day 4 of therapy in 54, 42, and 4 of these vignettes, respectively. Agreement between reviewers was high [Mean (±95% CI) intra-class correlation coefficient 0.86 (0.81-0.91)]. The sensitivity of the spectrum score method without PO credits to predict steward judgments of de-escalation events was only 56.7% (specificity 97.9%). Vignettes included in the final exercise were created as a subset of the initial 300 cases, and comparison of the mean (±SD) Likert scores for vignettes included in both the initial and final and exercises was 4.2(1.3) and 4.6 (1.1), respectively (*P* <0.003).

Taken together the vignette exercises suggest that inclusion of the route of administration was an important consideration in the assessment of de-escalation events, and that IV to PO conversion was viewed favorably when assessing de-escalation events. Table 4 summarizes the three refinement and validation exercise findings.

**Table 4:** **Validation and refinement of the spectrum score method to measure antimicrobial de-escalation**

**Validation exercise**	**Sensitivity (%)**	**Specificity (%)**	**Positive predictive value (%)**	**Negative predictive value (%)**	**Comments**
Convergent validation of original spectrum score	86.3	96.0	87.5	95.6	Reference standard de-escalation prevalence in vignettes was 24.2%. Route of administration data not included in cases. Mean Likert score was 0.51 points higher for cases where regimens contained ≥ 1 antimicrobial available in a PO dosage form administered on day 4 (p = 0.003).
Refinement exercise to verify that IV to PO conversion impacted expert opinion of de-escalation events	NA	NA	NA	NA	Mean Likert scores for regimens containing similar antimicrobials but differing routes of administration by day 4 was 5.0 (1.5) and 4.6 (1.5) for PO and IV cases (P = 0.002), respectively. Linear regression used to estimate an additional credit to the spectrum score ∆ to account for the group mean differences in Likert score associated with conversion from IV to PO therapy. A 6 point credit was added to spectrum score ∆ values for regimens with >50-100% PO and 3 point credit for regimens >0 but < 50% PO.
Convergent validation of spectrum score method including PO offsets	96.2	93.6	94.4	95.7	Reference standard de-escalation prevalence in vignettes was 53.0%. Route of administration data included in cases and PO credits applied to spectrum score ∆.

There were five discordant cases between the spectrum score with PO credits and reference method. Tetracyclines, which were only included in five vignettes, were involved in three of the discordant cases. Reviewers scored regimens in favor of de-escalation when tetracyclines were included on day 4 compared to the spectrum score method. Another discordant case involved the addition of a duplicative spectrum PO antibiotic to an IV regimen on day 4, which was classified an escalation by reviewers and a de-escalation by the spectrum score method due to the PO credit awarded. In the final discordant case, one of four antimicrobials was administered PO on day 2, but the antimicrobials remained identical on day 4 with two antimicrobials administered by the PO route. Experts scored this case as a de-escalation; however the case was not eligible for the credit as not all antimicrobials were administered IV on day 2.

## Discussion

The spectrum score method and findings are unique, and we are unaware of studies where definitions of antimicrobial de-escalation have been compared. Development of this approach was based in part on the opinions of antimicrobial stewards obtained during the Delphi process which enhanced construct validity. In the absence of a clear reference standard we compared the spectrum score method to that of antimicrobial stewards who have the most expertise and interest in the measurement of de-escalation events. A major finding of this investigation includes the high degree of agreement between the spectrum score predictions of de-escalation status and independent expert judgments applied at a patient-level which suggests convergent validity. A second major finding is the observation that antimicrobial stewards view IV to PO conversion favorably when classifying de-escalation events. Frequently clinicians make pragmatic tradeoffs when altering antimicrobial therapy in preparation for discharge which sometimes may be viewed as therapy simplification rather than verbatim selection of the most narrow-spectrum antimicrobial. While addition of the PO credits improved the sensitivity to detect de-escalation events classified by stewards, providing information on the route of antimicrobial administration to the experts in addition to the specific antimicrobials administered resulted in an increase in classification of de-escalation events in the vignettes. Further, the sensitivity to detect de-escalation events based on spectrum alone decreased when the route of administration information was added to the cases. The observation that stewards perceived tetracyclines as a relatively narrow-spectrum antimicrobial class despite susceptibility data to the contrary is also interesting. The reason behind this perception is unclear but may be related to the use of tetracyclines almost exclusively for coverage of atypical pathogens in treatment of pneumonia within the U.S., or a (subconscious) tendency to consider use of older drugs as a form of de-escalation.

Strengths of the method are that it was developed based upon the opinions of antimicrobial stewards and is anchored within the constructs of objective VA-wide microbial susceptibility data. The decision points of hospital day 2 and day 4 to measure baseline and follow-up therapy were supported as favorable time-points for measuring de-escalation events by antimicrobial stewards [7,46]. An additional strength of the method is that it can be adapted to classify antimicrobial de-escalation events in other electronic medical record systems.

Limitations of the method include reliance upon VA microbiology data to define spectrum score values. Antimicrobial susceptibilities and hence spectrum scores will likely change over time; which will require a regular re-evaluation of organism-antimicrobial susceptibilities. Further, in some instances VA susceptibility data were lacking and published susceptibility estimates were dated. It is possible that differences in susceptibility patterns across facilities may impact drug selection and de-escalation practice; however in an attempt to minimize these differences, we converted susceptibility estimates to ordinal values to facilitate generalization and organisms with MDR potential were weighted more heavily in accordance with Delphi panel preferences. The number of antimicrobial stewardship experts rendering judgments on de-escalation events was limited, and consensus was not obtained on all patient cases. In the final validation exercise, sensitivity of the method to detect de-escalation events in the vignettes was highly dependent on the addition of PO credits. It is important to remember that the estimates for de-escalation were generated from vignettes which were based upon antimicrobial regimens administered to patients in the cohort. In limited cases it was necessary to makes slight modifications of regimens to fit the vignette format, and it is unknown if the observed de-escalation rate reflects the actual de-escalation rate as measured in patients. However, the importance of PO conversion was identified in all three validation and refinement exercises. Conversely, disagreements between the spectrum score method and the expert judgments are to be expected, and lack of 100% agreement is not evidence for lack of validity.

Antimicrobial de-escalation and IV to PO conversion programs have been associated with reductions in length of hospitalization, inpatient antimicrobial use, adverse events, cost, and recovery of antimicrobial-resistant microbes [1,5,47-49]. However, few studies have clearly defined how de-escalation status was measured, and the quality of evidence in this area is poor [5,50]. The use of a limited scoring system based upon Gram-negative activity of anti-pseudomonal β-lactam and fluoroquinolone antibiotics has been previously described [51]. Recently, quality indicators for the management of antimicrobial use in sepsis have been described that recommend “changing to pathogen-directed therapy after culture-results become available” [52]. An IV to PO conversion quality measure has also been proposed for highly bioavailable antimicrobials in a clinically stable population; however we are unaware of recommendations that incorporate both concepts into a single measure [53].

Future work should include application and automation of the spectrum score method to measure antimicrobial de-escalation in electronic medical records. We are currently conducting such an analysis of VA-wide facility-level de-escalation rates in HCAP. Additional work should also include an assessment of the importance of PO therapy in the assessment of de-escalation therapy. Further, validation of the spectrum score method to estimate antimicrobial de-escalation in other patient populations, disease states, and electronic medical records systems is warranted.

## Conclusions

The spectrum score method exhibited excellent agreement with antimicrobial steward judgments of antimicrobial de-escalation events in pneumonia. The method, which is based upon the spectrum of antimicrobial regimens administered at subsequent time-points during hospitalization and the utilization of IV to PO conversion, can be applied to electronic medical records data to assess antimicrobial de-escalation in patients with healthcare associated pneumonia.

## Additional files

Additional file 1:
**Instructions on how to adapt the spectrum score method to measure antimicrobial de-escalation with electronic antimicrobial use data.**
Additional file 2:
**Ordinal Scores for Monotherapy and Combination Therapy Regimen-organism Pairs Represented in Vignettes.**

## Abbreviations

Spectrum Score ∆: Subtraction of the day 4 spectrum score value from the day 2 spectrum score value
PO: Oral
IV: Intravenous
VA: Veterans Healthcare Administration
HCAP: Healthcare associated pneumonia
NA: No intrinsic activity
ND: No VA susceptibility data available
CLSI: Clinical Laboratory Standards Institute
BCMA: Bar code medication administration
SD: Standard deviation 95%
CI: Ninety-five percent confidence intervals
