# Supplementary material for: Description and validation of a spectrum score method to measure antimicrobial de-escalation in healthcare associated pneumonia from electronic medical records data
Source: BMC Infect Dis. 2015 Apr 25;15:197. doi: 10.1186/s12879-015-0933-9 (PMC4418054; doi:10.1186/s12879-015-0933-9)
Supplement: Additional file 1: — Instructions on how to adapt the spectrum score method to measure antimicrobial de-escalation with electronic antimicrobial use data. [file 12879_2015_933_MOESM1_ESM.docx]

**Appendix A: Instructions on how to adapt the spectrum score method to measure antimicrobial de-escalation with electronic antimicrobial use data.**

Adaptation of the method will be best accomplished by use of Structured Query Language for managing data held in a relational database management system.

1) Patient-level antimicrobial use data needs for the population of interest should contain: the name and route of administration for each antimicrobial administered on each calendar day during hospitalization through at least day 5 for each admission. Each calendar day runs 24 hours from 00:00 through 23:59 military time. The spectrum score method is designed to measure de-escalation in patients hospitalized for more than 4 calendar days. Estimation of de-escalation therapy is limited to antimicrobials administered by the oral(PO) and intravenous(IV) routes and to antimicrobials which can be classified within the antimicrobial groupings reported in the manuscript Table 1 or in Table 4 of reference 7.

2) Patient-level daily antimicrobial regimen data should be “smoothed” prior to determining spectrum scores.

a. Prepare a table that provides a log of when antimicrobials were administered to each patient that includes the route of administration for each antimicrobial. All of these data must be standardized.

b. Create a variable for each entry that indicates the next time a specific antimicrobial is given to the same patient, as well as the time between the present dose and the next dose of the same drug (the gap).

c. Create a variable that flags the need for filling a gap of more than one but less than or equal to three days for aminoglycosides, amikacin, vancomycin, daptomycin, and fluoroquinolones.

d. Create a variable that flags when antimicrobial doses are given on the discharge day by joining to admission and discharge data tables.

e. Create a variable that identifies a difference of greater than three days between the present and the previous and next dose of any antimicrobial to identify the beginning and ending of treatment courses.

f. Create variables that identify the time of the next and previous doses of an antibiotic for each patient.

g. Use the variables in the prior step to identify different courses of the same drug when the difference in day is greater than three.

h. Mark antimicrobial days for deletion where all of the following are true:

- The day difference between the administration time of the current dose and the time of the previous dose of the same antimicrobial is between 0 and 2 ( i.e., the day is part of a course longer than one day)
- The day difference between the administration time of the current dose and the next dose is greater than 3 ( i.e., the day is at the end of a course)
- The day is not at the end of an antimicrobial treatment course
- There is a change in the antimicrobial regimen on the following day
- It is not the beginning of an antimicrobial treatment course
- The number of antimicrobials in the course is greater than one
- There was a change in antimicrobial regimen in the previous day

i. Create a table with the key being patient and calendar day that has a comma-delimited list of all unique antibiotics given on the same day.

3) A table of all smoothed antimicrobial regimens administered on calendar days 2 and 4 of hospitalization for the population of interest should then be generated.

4) Use step 3 to create a table of all antimicrobial regimen spectrum scores in the population of interest. A complete list of all feasible joint probability antimicrobial combinations is beyond the scope of this manuscript, ordinal scores for both monotherapy and combination therapy regimen-organism pairs represented in the 300 vignettes can be obtained from **Appendix B**.

To determine the spectrum scores for antimicrobial combination regimens not available in Appendix C, point estimates of susceptibility for organism domains and individual antimicrobials in the combination regimens are necessary to compute the joint probability of susceptibility for a particular combination regimen. Refer to the manuscript methods section for details on the approach to calculation of spectrum scores for combination antimicrobial therapy.

5) Next, ordinal scores for select antimicrobial regimen-organism pairs (both monotherapy and combination therapy) are weighted for intrinsic resistance potential. To weight the ordinal susceptibility scores multiply ordinal susceptibility values for organism-antimicrobial regimen pairs involving *Staphylococcus aureus, Enterococcus faecium, Escherichia coli, Klebsiella spp.,* and *Acinetobacter spp.* by a factor of 1.25, and *Pseudomonas aeruginosa* by a factor of 1.75.

6) To calculate each antimicrobial regimen spectrum score, sum the 14 organism domain ordinal scores to create a composite spectrum score. An example illustrates step 2 in Figure 1.

7) To apply the spectrum score method to measure de-escalation, line item patient-level data should be organized to include: smoothed antimicrobial regimen composite spectrum score administered on calendar day 2; percentage of antimicrobials (after smoothing) in regimen administered by the oral (PO) route on calendar day 2; smoothed antimicrobial regimen composite spectrum score administered on calendar day 4; and percentage of antimicrobials (after smoothing) in regimen administered by the PO route on day 4.

8) Calculate the spectrum score ∆ for each patient by subtracting the calendar day 4 antimicrobial regimen composite spectrum score from the calendar day 2 score. (i.e. Manuscript Figure 2c.)

9) For patients with 100% of antimicrobials in the smoothed regimen administered by the intravenous (IV) route on calendar day 2, add 6 spectrum score points to the spectrum score ∆ value if 50-100% of their antimicrobials in the regimen were administered by the PO route on day 4, or 3 spectrum score points to the spectrum score ∆ value if > 0 but < 50% of their antimicrobials in the regimen were administered by the PO route on day 4. (i.e. Manuscript Figure 2d).

Do not add any additional spectrum score points to the spectrum score ∆ cases were PO therapy was administered on calendar day 2 or cases where 100% of therapy was administered IV on calendar day 4.

10) A positive spectrum score ∆ after adjustment for PO therapy administered on calendar day 4 is suggestive that antimicrobial de-escalation has occurred.
